# Origin of the Reductive Tricarboxylic Acid (rTCA) Cycle-Type CO_2_ Fixation: A Perspective

**DOI:** 10.3390/life7040039

**Published:** 2017-10-23

**Authors:** Norio Kitadai, Masafumi Kameya, Kosuke Fujishima

**Affiliations:** 1 Earth-Life Science Institute, Tokyo Institute of Technology, Ookayama, Meguro-ku, Tokyo 152-8550, Japan; akameya@mail.ecc.u-tokyo.ac.jp (M.K.); fuji@elsi.jp (K.F.); 2 Department of Biotechnology, The University of Tokyo, Tokyo 113-8657, Japan; 3 Institute for Advanced Biosciences, Keio University, Tsuruoka, 997-0017, Japan

**Keywords:** acetyl-CoA, astrobiology, carbon assimilation, chemical evolution, metabolism, origin of life, pyruvate, thiamine pyrophosphate, thioester

## Abstract

The reductive tricarboxylic acid (rTCA) cycle is among the most plausible candidates for the first autotrophic metabolism in the earliest life. Extant enzymes fixing CO_2_ in this cycle contain cofactors at the catalytic centers, but it is unlikely that the protein/cofactor system emerged at once in a prebiotic process. Here, we discuss the feasibility of non-enzymatic cofactor-assisted drive of the rTCA reactions in the primitive Earth environments, particularly focusing on the acetyl-CoA conversion to pyruvate. Based on the energetic and mechanistic aspects of this reaction, we propose that the deep-sea hydrothermal vent environments with active electricity generation in the presence of various sulfide catalysts are a promising setting for it to progress. Our view supports the theory of an autotrophic origin of life from primordial carbon assimilation within a sulfide-rich hydrothermal vent.

## 1. Introduction

**Figure 1:**
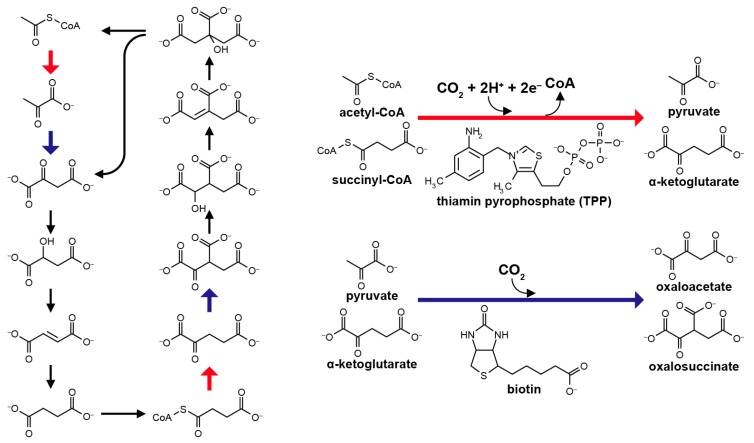
Structure of the reductive tricarboxylic acid (rTCA) cycle (**left**), in which the CO_2_ fixations leading to the pyruvate and α-ketoglutarate formations are mediated by thiamin pyrophosphate (TPP), whereas to the oxaloacetate and oxalosuccinate formations are by biotin (**right**).

The non-enzymatic processing of the reductive tricarboxylic acid (rTCA) cycle-type carbon assimilation has been among the most challenging themes in the field of the origin of life [1,2,3,4]. Various abiotic mechanisms to realize the reaction have been proposed, including the pyruvate formation from carbon monoxide (CO) and cyanide anion (CN^−^) in the presence of Ni^2+^ [5], a high pressure condensation of alkyl thiols and formic acid to pyruvate catalyzed by FeS [6], and the photo-electrochemical CO_2_ reduction and fixation into rTCA compounds on ZnS colloidal semiconductor under UV irradiation [7,8,9]. However, their contributions to life’s origin have been questioned [10] because large discrepancies exist between the proposed mechanisms and the corresponding metabolic processes. In the biological rTCA cycle, CO_2_ fixation is operated by the two enzyme cofactors (Figure 1): thiamine pyrophosphate (TPP) assists the conversion of acetyl-CoA to pyruvate and succinyl-CoA to α-ketoglutarate [11,12], whereas biotin mediates the formations of oxaloacetate and oxalosuccinate from pyruvate and α-ketoglutarate, respectively [13,14]. The two cofactors have been deduced to participate in autotrophic metabolism from the very beginning of the life’s evolution, at least from the stage of the last universal common ancestor (LUCA) that could have lived in deep-sea hydrothermal systems [15]. Remarkably, replacement of heteroatoms in their ring structures with others (e.g., O or N vs. S) does not inactivate, or in some cases even improves, their functional properties [16,17,18]. Various heterocyclic compounds with structural features resembling the two have been synthesized under simulated primitive environmental conditions [19,20,21]. Therefore, an alternative possibility is that prebiotic analogs of TPP and biotin with simpler structures that are initially formed via inorganic processes, facilitated the primordial carbon fixation that preceded the origin of life, were incorporated into proto-enzymes in the course of functional evolution, and eventually developed into the modern counterparts.

In this manuscript, we discuss the feasibility of this scenario with a special attention to the second part; the non-enzymatic cofactor-assisted CO_2_ fixation. Our study focused on the acetyl-CoA conversion to pyruvate on TPP because thiolated acetate derivatives (thioacids; R-COSH, thioesters; R-COS-R’), plausible ancient forms of acetyl-CoA [22], were possibly present on the primitive Earth [23,24]. Although recent geochemical surveys of the present-day submarine hydrothermal fields observed no evidence of their abiotic formations [25,26,27], the results do not necessarily deny their presence in the Hadean ocean hydrothermal ones because the geological situations are likely largely different from each other. For instance, it has been shown that a high-temperature basalt–seawater interaction in a CO_2_-rich condition results in the increase of solution pH to highly alkaline (pH ≥ 12; [28,29]). Owing to a denser distribution of metals in the ancient deep-ocean [30,31] derived from much greater hydrothermal activity than the present level [32], the alkaline fluid–seawater mixing in the early basalt-hosted hydrothermal systems could have precipitated metal sulfides as the main body of hydrothermal mineral deposits [33]. This environmental setting favors the abiotic production of thioester [24]. The thioester/thioacid conversion to pyruvate corresponds to the initial step of the rTCA cycle. Thus, no development of the subsequent proto-metabolism is expected unless an effective geochemical route to the pyruvate formation was established. Citrate can be a source of oxaloacetate and pyruvate [34], but a proposed abiotic synthesis of citrate requires pyruvate [35]. Note that a simple heating of thioacids and thioesters in water in a range of temperature and pH results in the hydrolysis of the thioester bond [36,37], and no experimental evidence has been reported for the mineral-promoted CO_2_ fixation into them in the prebiotic context [38], although approximately three decades have passed since the possibility was first proposed [1,39]. These facts motivated us to search the organic catalysts for the initiation of the primordial carbon assimilation.

## 2. Energetics of Pyruvate Synthesis

**Figure 2:**
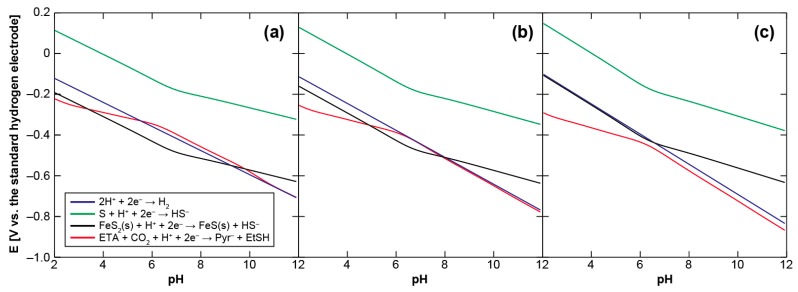
Eh-pH relationships of the pyruvate formation from ethylthioacetate and CO_2_ (red) and of the H_2_/H^+^ (blue), H_2_S/S (green) and mackinawite/pyrite (black) redox couples at (**a**) 25, (**b**) 60, and (**c**) 100 °C. See text and Appendix A for the calculation conditions and procedures.

**Figure B1:**
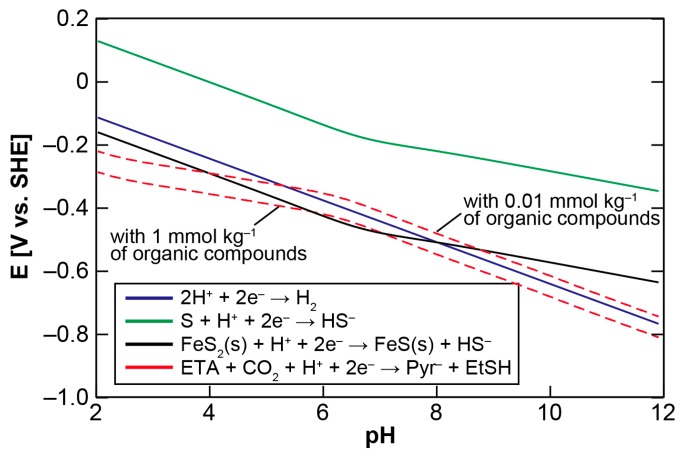
Figure 2b was re-calculated with the organic compounds’ concentrations of 1 mmol·kg^−1^ and 0.01 mmol·kg^−1^.

We initially examine the energetics of pyruvate synthesis using ethylthioacetate (ETA) as a prebiotic counterpart of acetyl-CoA to clarify the environmental condition necessary for it to be driven thermodynamically. Figure 2 shows the calculated Eh-pH relationship of the pyruvate formation (ETA + CO_2_ + H^+^ + 2e^−^ → pyruvate + ethanethiol (EtSH)), together with those of the H_2_/H^+^, H_2_S/S and mackinawite/pyrite (FeS/FeS_2_) redox couples, at 25, 60, and 100 °C (see Appendix A for the calculation procedure). S (solid sulfur) is used as the H_2_S oxidation product because the H_2_S/S redox couple provides a major potential control in the sulfide-rich hydrothermal vent environments [40]. The concentrations of H_2_ and H_2_S were assumed to be 1 mmol·kg^−1^, whereas that of CO_2_ to be 20 mmol·kg^−1^. 1 mmol·kg^−1^ is the representative H_2_ and H_2_S concentrations in the serpentine-hosted hydrothermal systems on land [41] and on the ocean-floor [42] that have been argued to be the most plausible settings for the origin of life [33,43,44], whereas 20 mmol·kg^−1^ corresponds to the steady-state CO_2_ concentration in the early ocean [45,46]. For organic compounds, 0.1 mmol·kg^−1^ was arbitrarily chosen because of no definitive constraint; calculations with different initial settings (Figure B1) showed that higher organics’ concentrations result in slightly lower Eh values.

It can be seen in Figure 2 that H_2_S does not generate the potentials required to drive the pyruvate formation over the examined aqueous conditions, while the lines of the H_2_/H^+^ and FeS/FeS_2_ redox couples intersect with the threshold. At 25 °C, the H_2_ and FeS oxidations provide favorable conditions for the CO_2_ fixation at pH 5.5–10.5 and 3–10, respectively. The pH ranges gradually shrink at higher temperature owing to the negative shift of the necessary potential with an increasing temperature. H_2_ loses its thermodynamic advantage at around 60 °C (Figure 2b), whereas FeS does at around 100 °C (Figure 2c). The H_2_ and FeS-driven pyruvate syntheses are therefore energetically possible only in a cool to warm and near neutral aqueous solution. Plausible conditions for the accumulation of pyruvate to a proto-metabolically significant extant may be further restricted by the unstable character of pyruvate in acidic pH [47].

## 3. TPP-Assisted Pyruvate Synthesis: A Mechanism

Then, is pyruvate producible non-enzymatically under sufficiently reductive conditions, such as nearby a H_2_-rich hydrothermal vent discharging FeS precipitate continuously, aided by TPP or its prebiotic analogs? Note that the direct coupling of FeS oxidation with CO_2_ reduction and fixation is unlikely to occur due to the high activation energy even when the overall process is thermodynamically favorable [48].

**Figure 3:**
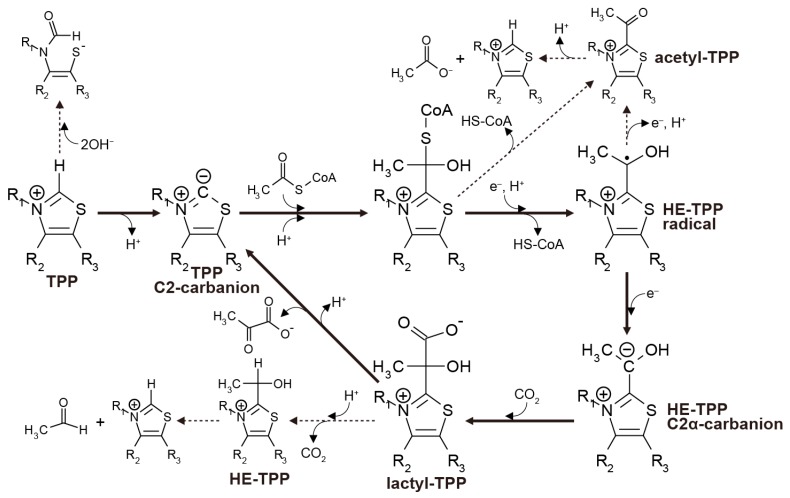
Thiamin pyrophosphate (TPP)-assisted pyruvate formation operated in pyruvate:ferredoxin oxidoreductase (PFOR) (solid arrows) illustrated on the basis of the reported models [11,39,40,41] and the side reactions that disrupt the overall process (dashed arrows).

In the biological rTCA cycle, pyruvate synthesis is catalyzed by an iron-sulfur enzyme called pyruvate:ferredoxin oxidoreductase (PFOR), or 2-oxoacid:ferrdoxin oxidoreductase (OFOR) [11,49,50,51]. The catalytic center of all the known enzymes contains TPP as an essential cofactor for the one-carbon transfer. The process starts with the deprotonation of the C2 carbon in the thiazolium ring, followed by the nucleophilic attack of the resulting carbanion on the carbonyl carbon of acetyl-CoA to form a tetrahedral intermediate (Figure 3). The intermediate then undergoes CoA release, and one electron transfer from a reduced iron-sulfur cluster yields the hydroxyethyl-TPP (HE-TPP) radical. A second electron addition reduces it to the HE-TPP C2α carbanion, and its nucleophilic attack to CO_2_ makes lactyl-TPP that finally produces pyruvate.

The stability and reactivity of the reaction intermediates have been examined using TPP that is unbound to enzymes. The proton dissociation from the thiazolium C2 position occurs with the pK_a_ of 17–19 [52], while alkaline pH (>9.40) favors the opening of the thiazolium ring [53]. Acetyl-TPP, a one-electron oxidation product of the HE-TPP radical [54], hydrolyzes rapidly to acetate and TPP at neutral and alkaline pH (e.g., t_1/2_ = 58 s at pH 7.0 and 24 °C [55]). The pyruvate release from lactyl-TPP competes with the decarboxylation of lactyl-TPP to HE-TPP; the decarboxylation predominates at pH < 9.5 (25 °C) [56]. When the sulfur atom in the thiazolium ring is replaced with nitrogen, it increases the stability against ring-opening, while it suppresses the ylide formation [16].

In PFOR and OFOR, a conserved Glu residue stimulates the deprotonation of the thiazolium C2 at the TPP-binding site with a low dielectric constant (ε_r_ = 13–15 [57]). Electrons are provided by [4Fe-4S] ferredoxins and are transported from the external enzyme surface to the catalytic center via optimally arranged single or multiple [4Fe-4S] cluster(s) [11]. The proximal [4Fe-4S] cluster that locates within 15 Å from TPP [58,59,60] allows for rapid electron transfer to the adducts of TPP immediately after they are formed, thereby prohibiting the intermediates from decaying. Although the bacterial and archaeal enzymes differ in the subunit composition and overall structure, the proximal [4Fe-4S] cluster is conserved [60], indicating the importance of this electron transfer pathway in the enzymatic processes. The [4Fe-4S] cluster possesses the potential as low as −540 mV (vs. standard hydrogen electrode; SHE [61]) that is sufficiently low to drive the energetically up-hill acetyl-CoA carboxylation.

## 4. Discussion: Feasibility of Abiotic Pyruvate Synthesis in a Geological Setting

The above summary clearly indicates that the TPP-assisted pyruvate formation never takes place in single aqueous condition. In contrast, at the mineral-water interface with a low dielectric constant (ε_r_ = 26–53 [62]), the thiazolium ylide and the HE-TPP carbanion are expected to be stabilized significantly [57], while such condition accelerates the decarboxylation of lactyl-TPP [56,63]. It has been shown that imidazolium species, which contain nitrogen atom at the place of sulfur in the thiazolium structure, effectively catalyze the CO_2_ reduction to CO and formate on FeS_2_, and to ethylene glycol on gold under an externally applied negative electric potential (at −0.85 V (vs. SHE)) [64,65]. The CO_2_ activation was inferred to be induced by the imidazolium ylide formation on the negatively charged electrodes, followed by the CO_2_ binding at the C2 position [64,65]. FeS could also provide reactive surface and electric energy by coupling with its oxidation to FeS_2_, as was demonstrated for the H_2_S reduction to H_2_ [66,67], nitrogen oxides to ammonia [68,69], ethyne to ethane, and ethane [70], and the reductive amination of α-keto acids [71]. Interestingly, freshly precipitated FeS has the point of zero charge (pH_ZPC_) of around 7.5 [72], whereas the pH_ZPC_ of FeS_2_ is around 1.5 [73,74]. FeS is thus expected to provide a wide range of surface pH in the course of its oxidation even under a constant aqueous condition, and controls the speciation of adsorbed molecules [75,76,77]. A drawback of the FeS-driven CO_2_ fixation is that the electron supply ceases when the FeS surface is fully oxidized. Organic molecules thus need to be transported onto fresh FeS via diffusion and/or convection to continue their reductions.

**Figure 4:**
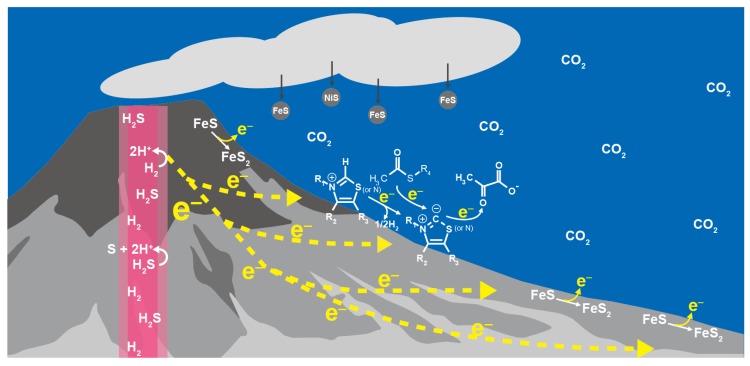
Geo-electrochemical pyruvate formation in the early ocean hydrothermal vent environment as a possible initial step of the primordial carbon fixation.

Wider and diverse electrochemical environments are available in sulfide-rich hydrothermal systems on the ocean floor [78], where the potential gradient between the hydrothermal fluids and seawater across the sulfide deposits drives the flow of electric current, and promotes redox reactions at the vent-seawater interface by the continuous electron supply in the presence of various mineral catalysts (Figure 4) [40,79,80]. If 1 mmol kg^−1^ H_2_ in hot and alkaline hydrothermal fluids serves as the electron source, it generates the potential (e.g., −0.84 V (vs. SHE) at 100 °C and pH 12) well below the desired value for the pyruvate synthesis at 25 °C and slightly acidic to neutral pH (−0.3~−0.4 V (vs. SHE); Figure 2). Water molecules in an external electric field have a low dielectric property [81,82]. The geo-electrochemical setting thus could provide reaction conditions resembling the electron transfer system in PFOR and OFOR in terms of the direct donation of low-potential electrons from metal-sulfur clusters to the catalytic center with a low dielectric constant.

The hydrothermal setting also favors the abiotic amino acid synthesis [83,84] and polymerization [19,85,86,87]. Amino acids and short peptides in some cases improve the stability and activity of electrocatalysts [88,89,90,91]. Peptides with 10–20 monomers long have the capability of recognizing TPP [92] and biotin [93]. These evidences may imply an early-stage interaction of peptides and cofactors near the vent surface that could have played a positive role in the selective and efficient progress of the primordial carbon assimilation [94,95,96]. A conclusion for the abiotic origin of the TPP-mediated pyruvate synthesis must await the time when the aforementioned possibilities are experimentally tested under simulated primordial geo-electrochemical conditions. As a future experimental study, it is of particular importance to examine whether prebiotically producible heterocyclic compounds with structural features resembling TPP [19,20,21] can assist the CO_2_ fixation in the proposed environment. The pyrimidine and pyrophosphate parts of TPP may be replaced with simpler structures (e.g., –CH_3_) without deactivation, and imidazolium and perhaps oxazolium rings could serve as electron carriers in a manner similar to the thiazolium one in TPP. If primitive analogs of TPP can catalytically provide pyruvate in a geological setting, the situation will also favor the C4 to C5 conversion (i.e., succinyl-CoA → α-ketoglutarate; Figure 1). Although extant organisms employ two distinct enzymes for the pyruvate and α-ketoglutarate syntheses, the primordial system could have used a single catalyst for the two reactions and have later developed the optimally-tuned enzymes for each, as was proposed for the evolution of many enzymes [97]. For the other CO_2_ fixation steps (pyruvate → oxaloacetate, α-ketoglutarate → oxalosuccinate; Figure 1), the energetically most difficult process is the tautomerization of reactants from the keto to the enol forms [98]. This problem may be overcome by borate [10,99] given the substantial amounts of boron released into ocean in the course of the early submarine hydrothermal activities (1.8–4.5 × 10^10^ mol·year^−1^ [100]) and its accumulation on seafloor clay minerals [101]. Alternatively, there is a possibility that the TPP and biotin-mediated system is a genuine biological invention with no relic of the relevant prebiotic processes [102]. Without these cofactors, no effective CO_2_ fixation through the rTCA cycle is expected, and thus, the early autotrophs would have had completely different metabolic strategies from those as we know [103]. In either case, the origin of life scenario must connect smoothly the current and progressively updated knowledge of the ancient geochemistry and biochemistry [104].

Finally, we discuss the suitability of other inorganic carbon compounds than CO_2_ as a precursor for the abiotic pyruvate production. The reaction could be facilitated in the presence of aldehydes because the usage of acetaldehyde instead of acetyl-CoA skips the route of the unstable HE-TPP radical formation [105]. Aldehydes also serve as a carbon source of thioesters through the oxidative coupling with thiols [106,107], and the reaction is catalyzed by thiazolium compounds [108]. However, the availability of aldehydes in the early-ocean hydrothermal systems remains controversial [109,110,111]. Formate may be a more realistic C1 source because formate has occasionally been observed in the present-day hydrothermal systems with high concentrations of up to ~0.7 mM [27,112,113]. Actually, the enzyme “pyruvate formate-lyase (PFL)” catalyzes the reversible conversion of pyruvate and CoA into acetyl-CoA and formate; the system plays a central role in anaerobic glucose fermentation in diverse Eukarya and Bacteria [114,115]. A drawback of the formate fixation is that it is a highly thermodynamically up-hill reaction (the standard Gibbs energy of reaction (∆*_r_G^o^*) = ~−21 kJ·mol^−1^; [116]) with the equilibrium constant of 2 × 10^−4^. The net PFL reaction is neither oxidation nor reduction; hence the energy barrier cannot be overcome by the geo-electrochemical mechanism discussed above. The low reactivity of formate, which is a much poorer electrophile than CO_2_ [117], is another problem to be solved. PFL activates the formate condensation by a radical mechanism using two cysteine and one glycine residues as radical carriers [118,119,120]. It is unclear whether such a radical process can be operated non-enzymatically in water or on minerals with the aid of prebiotically available short peptides. 

## 5. Concluding Remarks

Abiotic CO_2_ fixation is among the most fundamental steps for life to originate, but no geochemically feasible process that drives the reaction has been acknowledged [121]. If the above-discussed mechanism occurs with prebiotically producible cofactor analogs, favorable conditions for it to progress could have distributed widely on the early ocean floor because the global thermal convection at that time is considered to be much greater than the present level [32]. It can also be envisioned that the geochemical CO_2_ fixation is a common phenomenon on terrestrial planets and satellites because hydrothermal activity is widespread in our solar system including on Europa, Enceladus, and the ancient Mars [122,123,124]. Future experimental study that mimics the conditions of the proposed model is expected to provide insights into the universality of autotrophic metabolism and its underpinning life systems in the cosmos.

## Appendix A

The Eh-pH relationship of the ethylthioacetate conversion to pyruvate, and of the H_2_/H^+^, H_2_S/S and FeS/FeS_2_ redox couples (Figure 2) were calculated, respectively, using the following equations:

(A1) $$E_{h}=\frac{-1}{2F}\left(\Delta_{f}G^{o}\left(H_{2}\right)+RT\alpha_{H_{2}}-2RTln\alpha_{H^{+}}\right),$$

(A2) $$E_{h}=\frac{-1}{2F}\left(x\left(\Delta_{f}G^{o}\left(H_{2}S\right)+RTln\alpha_{H_{2}S}\right)+\left(1-x\right)\left(\Delta_{f}G^{o}\left(HS^{-}\right)+RTln\alpha_{HS^{-}}\right)-\;\Delta_{f}G^{o}\left(S\right)-\left(1+x\right)RTln\alpha_{H^{+}}\right),$$

(A3) $$E_{h}=\frac{-1}{2F}\left(\Delta_{f}G^{o}\left(FeS\right)+x\left(\Delta_{f}G^{o}\left(H_{2}S\right)+RTln\alpha_{H_{2}S}\right)+\left(1-x\right)\left(\Delta_{f}G^{o}\left(HS^{-}\right)+\;RTln\alpha_{HS^{-}}\right)-\Delta_{f}G^{o}\left(FeS_{2}\right)-\left(1+x\right)RTln\alpha_{H^{+}}\right),$$

and

(A4) $$\begin{array}{c} E_{h}=\frac{-1}{2F}(y\left(\Delta_{f}G^{o}\left(Pyr\right)+RTln\alpha_{Pyr}\right)+\left(1-y\right)\left(\Delta_{f}G^{o}\left(Pyr^{-}\right)+RTln\alpha_{Pyr^{-}}\right)+\\ z\left(\Delta_{f}G^{o}\left(EtSH\right)+RTln\alpha_{EtSH}\right)+\left(1-z\right)\left(\Delta_{f}G^{o}\left(EtS^{-}\right)+RTln\alpha_{EtS^{-}}\right)+\\ \left(1-n\right)\Delta_{f}G^{o}\left(H_{2}O\right)-\left(\Delta_{f}G^{o}\left(ETA\right)+RTln\alpha_{ETA}\right)-n\left(\Delta_{f}G^{o}\left(CO_{2}\right)+RTln\alpha_{CO_{2}}\right)-\\ m\left(\Delta_{f}G^{o}\left(HCO_{3}^{-}\right)+RTln\alpha_{HCO_{3}^{-}}\right)-\left(1-n-m\right)\left(\Delta_{f}G^{o}\left(CO_{3}^{2-}\right)+RTln\alpha_{CO_{3}^{2-}}\right)-\\ \left(2+y+z-m-2n\right)RTln\alpha_{H^{+}}). \end{array}$$

In these equations, *T*, *R*, and *F* stand for temperature in kelvin, the gas constant (8.31447 J·mol^−1^·K^−1^), and the Faraday constant (96,485 J mol^−1^ V^−1^), respectively. *a_i_* represents the activity of the species *i*. *x*, *y*, *z*, *n*, and *m* signify the mole fraction of H_2_S ($=\frac{M_{H_{2}S}}{M_{H_{2}S}+M_{{\mathrm{HS}}^{-}}}$), pyruvic acid ($=\frac{M_{\mathrm{Pyr}}}{M_{\mathrm{Pyr}}+M_{{\mathrm{Pyr}}^{-}}}$), ethanethiol ($=\frac{M_{\mathrm{EtSH}}}{M_{\mathrm{EtSH}}+M_{{\mathrm{EtS}}^{-}}}$), CO_2_ ($=\frac{M_{{\mathrm{CO}}_{2}}}{M_{{\mathrm{CO}}_{2}}+M_{{\mathrm{HCO}}_{3}^{-}}+M_{{\mathrm{CO}}_{3}^{2-}}}$), and HCO_3_^−^ ($=\frac{M_{{\mathrm{HCO}}_{3}^{-}}}{M_{{\mathrm{CO}}_{2}}+M_{{\mathrm{HCO}}_{3}^{-}}+M_{{\mathrm{CO}}_{3}^{2-}}}$), respectively (*M_i_* denotes the molality of the species *i*). In addition, ∆*_f_G^o^(i)* represents the standard molal Gibbs energy of formation of the species *i* at desired temperature, which were calculated according to the revised HKF equations of state [125] together with the thermodynamic data and the revised HKF parameters reported in [126] for H_2_ and H_2_S, in [127] for HS^−^, in [128] for ethylthioacetate, pyruvate and pyruvic acid, and in [129] for ethanethiol. The ∆*_f_G^o^* value for ethanethiol anion (EtS^−^) was estimated using the value of ∆*_f_G^o^* for ethanethiol in combination with its ionization constant as a function of temperature [130]. The temperature dependences of ∆*_f_G^o^* for S (solid sulfur) and FeS_2_ were calculated as follows:

(A5) $$\Delta G_{P,T}^{o}=\Delta G_{P_{r},T_{r}}^{o}-S_{P_{r},T_{r}}^{o}\left(T-T_{r}\right)+\int_{T_{r}}^{T}C_{P_{r}}^{o}dT-T\int_{T_{r}}^{T}C_{P_{r}}^{o}dlnT+\int_{P_{r}}^{P}V_{T}^{o}dP$$

where $\Delta G_{P_{r},T_{r}}^{o}$ and $S_{P_{r},T_{r}}^{o}$, respectively, represent the standard molal Gibbs energy and entropy at the reference temperature (*T_r_* = 298.15 K) and pressure (*P_r_* = 1 bar). $C_{P_{r}}^{o}$ represents the standard molal heat capacity at *P_r_*, and $V_{T}^{o}$ denotes the standard molal volume at the temperature of interest. In the present calculation, the values of $\Delta G_{P_{r},T_{r}}^{o}$, $S_{P_{r},T_{r}}^{o}$, and $C_{P_{r}}^{o}$ as a function of temperature for S and FeS_2_ were taken from [131] and [132], respectively, while the value of $V_{T}^{o}$ was assumed to be constant in the range of temperature of our interest. The ∆*_f_G^o^* for FeS was estimated from the equilibrium constant of FeS dissolution (FeS + 2H^+^ → Fe^2+^ + H_2_S [133]) together with the ∆*_f_G^o^* for Fe^2+^ (referred from [127]) and for H_2_S. The values of *x*, *y*, *z*, *n*, and *m* are expressed, respectively, as:

(A6) $$x=\frac{\gamma_{HS^{-}}a_{H^{+}}}{\gamma_{HS^{-}}a_{H^{+}}+\gamma_{H_{s}S}K_{H_{s}S}},$$

(A7) $$y=\frac{\gamma_{Pyr^{-}}a_{H^{+}}}{\gamma_{Pyr^{-}}a_{H^{+}}+\gamma_{Pyr}K_{Pyr}},$$

(A8) $$z=\frac{\gamma_{EtS^{-}}a_{H^{+}}}{\gamma_{EtS^{-}}a_{H^{+}}+\gamma_{EtSH}K_{EtSH}},$$

(A9) $$n=\frac{\gamma_{HCO_{3}^{-}}\gamma_{CO_{3}^{2-}}a_{H^{+}}^{2}}{\gamma_{HCO_{3}^{-}}\gamma_{CO_{3}^{2-}}a_{H^{+}}^{2}+\gamma_{CO_{2}}\gamma_{CO_{3}^{2-}}K_{CO_{2},1st}a_{H^{+}}+\gamma_{CO_{2}}\gamma_{HCO_{3}^{-}}K_{CO_{2},2nd}},$$

and

(A10) $$m=\frac{\gamma_{CO_{2}}\gamma_{CO_{3}^{2-}}K_{CO_{2},1st}a_{H^{+}}}{\gamma_{HCO_{3}^{-}}\gamma_{CO_{3}^{2-}}a_{H^{+}}^{2}+\gamma_{CO_{2}}\gamma_{CO_{3}^{2-}}K_{CO_{2},1st}a_{H^{+}}+\gamma_{CO_{2}}\gamma_{HCO_{3}^{-}}K_{CO_{2},2nd}}.$$

Therein, *γ_i_* represents the activity coefficient of the species *i* (*a_i_* = *M_i_* × *γ_i_*) and *K_i_* the dissociation constant of *i* (*i* → *i^−^* + H^+^), whose values were calculated as:

(A11) $$K_{i}=exp\left(\frac{\Delta_{f}G^{o}\left(i\right)-\Delta_{f}G^{o}\left(i^{-}\right)}{RT}\right)$$

for H_2_S, pyruvic acid, and ethanethiol, and as:

(A12) $$K_{CO_{2},1st}=exp\left(\frac{\Delta_{f}G^{o}\left(HCO_{3}^{-}\right)-\Delta_{f}G^{o}\left(CO_{2}\right)-\Delta_{f}G^{o}\left(H_{2}O\right)}{RT}\right)$$

and

(A13) $$K_{CO_{2},2nd}=exp\left(\frac{\Delta_{f}G^{o}\left(CO_{3}^{2-}\right)-\Delta_{f}G^{o}\left(CO_{2}\right)-\Delta_{f}G^{o}\left(H_{2}O\right)}{RT}\right)$$

for CO_2_. The ∆*_f_G^o^* for H_2_O was referred from [134]. In all calculations, the values of *γ_i_* were calculated with the extended Debye–Hückel equation [135] setting the ionic strength to be 0.1 (NaCl). The pressure was set to 1 bar. S^2−^ and the ion pair NaHS were not considered in this calculation because these are expected to be minor S and/or Na species in the examined aqueous conditions [136,137].

## Appendix B

See Figure B1.
